# Suicidal Thoughts and Behaviors Among Transgender Adults in Relation to Education, Ethnicity, and Income: A Systematic Review

**DOI:** 10.1089/trgh.2019.0009

**Published:** 2019-10-16

**Authors:** Noah J. Adams, Ben Vincent

**Affiliations:** ^1^ Department of Leadership, Higher and Adult Education, Ontario Institute for Studies in Education, University of Toronto, Toronto, Canada.; ^2^ Department of Sociology, University of York, York, United Kingdom.

**Keywords:** gender identity, health disparities, mental health, minority stress, suicide, transgender/transsexual

## Abstract

**Introduction:** This systematic review assessed the impact of race/ethnicity, education, and income on transgender individual's lifetime experience of suicidal thoughts and behaviors (SITB) in gray and published literature (1997–2017).

**Methods:** Sixty four research projects (108 articles) were identified in WorldCat, PubMed, and Google Scholar. Articles were included if they were published in Canada or the United States, included original quantifiable data on transgender SITBs, and had ≥5 participants, at least 51% of whom were ≥18 years.

**Results:** Across all projects suicide ideation averaged 46.55% and attempts averaged 27.19%. The majority of participants were Caucasian, whereas the highest rate of suicide attempts (55.31%) was among First Nations, who accounted for <1.5% of participants. Caucasians, by contrast, had the lowest attempt rate (36.80%). More participants obtained a bachelor's degree and fewer an associate or technical degree than any other level of education. Suicide attempts were highest among those with ≤some high school (50.70%) and lowest among those with an advanced degree (30.25%). More participants made an income of $20–$50,000/year and less $10–$20,000 than any other income bracket.

**Conclusion:** SITBs, among the transgender population, are both universally high and impacted by race/ethnicity, educational attainment, and income. These findings may be useful in creating culturally and factually informed interventions for transgender individuals experiencing SITBs and in informing future research on this topic.

## Introduction

Transgender people—whose gender identity is incongruent with the sex assigned to them at birth—are increasingly publicly visible.^1^ Despite, or perhaps because of this, they are extremely vulnerable to discrimination, violence, and marginalization, the experience of which has extremely negative impacts on their health and well-being.^2^ Shockingly high rates of suicidal thoughts and behaviors (SITBs), previously identified as averaging 55% for ideation and 29% for attempts over the lifetime, are a clear indication of these negative impacts.^3^ What relationship do race/ethnicity, educational attainment, and income have with SITBs among transgender adults? We address this question through a systematic review of 21 years of peer-reviewed and gray literature on the topic and, in doing so, update and provide an added analysis to the lead author's previous publication on the matter.^3^ To do so, we used data on transgender participants from 64 distinct research projects^i^ published in 108 articles between 1997 and 2017 (Table 1).^4–110^

**Table 1.** Studies in This Meta-Synthesis

No.	Author/s	#	Ideation	Attempt	Description
1	Cole et al.^4^	435		Ever: 14.48%	Analyzed incidence of Axis 1 or 2 diagnoses through retrospective chart review of self-diagnosed transsexuals who presented to Texas gender clinic since 1980. Charts included information from 1 to 2 h clinical interviews, biographical and medical questionnaires and, in some cases, psychometric inventories. The previous publication on this dataset^3^ reported the time period as “before treatment.” This was changed to “ever” as no patients had GRS at data collection and, in any case, none attempted after GRS. The attempt rate was also changed from 15% to 14.48% to maintain consistency in reporting findings to two decimal places wherever possible
2	Devor^5,6^	45	Ever: 28.89%	Ever: 22.22%	Investigated life experiences of FTM individuals using qualitative interviews conducted from 1998 to 2002. Suicidality information volunteered during the interview. This is the first needs assessment conducted on the topic of transgender health
3	Rehman et al.^7^	28	After surgery: 7.14%		Explored post-GRS sex and surgery satisfaction among MTF patients of a New York City Hospital that received GRS from 1980 to 1994, using a mail-in questionnaire and, in some cases, interviews. Only information on the postsurgical period is reported, although it was noted that there was a marked decrease in attempts postsurgery
4	Mathy^8^	73^a^	Ever: 37%	Ever: 23.30%	Examined transgender suicidality among respondents to two human sexuality surveys on MSNBC website over 1 month (2000). First was selected random sample and second invited every thousandth. Given choice between male, female, and transgender, 0.2% of each sample selected the latter and were psychosocially matched to cis male and cis female controls and lesbian and gay comparison groups
5	Singer et al.,^9^ Kenagy^10,11^	103		Ever: 38.83%	Needs assessment that explored physical and mental health among Philadelphia-area transgender individuals for approximately a year (1996–1997)
6	Kenagy and Bostwick^12^	111	Ever: 62.16%	Ever: 26.12%	Community-based needs assessment that explored health and social service needs of Chicago transgender individuals recruited over 6 months (2000–2001). Replicated Singer et al.^9^ and Kenagy.^10,15^ Suicidality findings here amend those published in Adams et al.^3^ to correct for small rounding error
7	Bockting, et al.,^13,14^	181	Past 3 years: 52%		Analyzed the impact of a Minnesotan sexual health seminar on LGBT health-risk factors by following a cohort through an intervention using a case–control element to compare transgender and cis gender respondents. All participants in the 8 seminars conducted from 1997 to 2002 were asked to complete a survey pre and postintervention and after 3 months
8	Kenagy^10,11,15^	81	Ever: 46.91%	Ever: 19.75%	Needs assessment that investigated health and social service needs of a Philadelphia-area transgender community using data collected over 6 months (1997)
9	Risser et al.^16^	67^a^	Ever: 58.21%, past 30 days: 16.42%	Ever: 29.85%	Community-based needs assessment that investigated the sexual health risks and social and sexual health status of a group of transgender women in Houston over 2 months (2002–2003)
10	Xavier,^17^ Xavier and Simmons,^18^ Xavier et al.^19^	252^a^	Ever: 34.92%	Ever: 16.27%	Community-based needs assessment that investigated the health and social service needs of transgender people in Washington, DC recruited over 4 months (1999–2000)
11	Clements et al.,^20^ Clements-Nolle et al.,^21,22^	515		Ever: 32.23%	Examined HIV, risk behaviors, mental health, and health care use of transgender individuals in San Francisco over 5 months in 1997
12	Zians^23^	136	Past year: 31.62%	Ever: 17.65%	Community-based needs assessment of the health care and social service needs of transgender individuals in San Diego conducted over 7 months in 2004
13	Taylor^24,25^	73^a^	Ever: 54%	Ever: 28%	Community-based needs assessment conducted over 6 months in 2006 on transgender Manitoban and Northwestern Ontarian health and social service needs. As two-spirit encompasses Aboriginal LGB and/or transgender individuals there may be a small number of non-transgender respondents in the dataset. Participants given a choice of long or short-form questionnaire and survey party held for aboriginals
14	Landers and Gilsanz^26^	52	Past year: 30.77%		Survey conducted for the Massachusetts Department of Public Health over 10 days in 2009 using respondents from MassEquality's e-mail list to explore the impact of the State's equal marriage law on LGBT health and security. Perhaps because of a short data collection period, the response rate was 4.2%. Transgender respondents were also forced to select either LGB or transgender and thus likely under sampled
15	McDuffie and Brown^27^	70	Ever: 48.57%	Ever: 8.57%	Chart review of US Veterans examined for “gender identity disturbances” by second author at Tennessee VA office from 1987 to 2007. Study likely has great deal of participant overlap with Blosnich et al.,^41^ Brown et al.,^65^ and Bukowski et al.^110^ In contrast with Adams et al.^3^ the denominator used in our analysis includes those with missing ideation data.
16	Nuttbrock et al.^28^	571	Ever: 53.50%	Ever: 27.90%	Explored psychiatric impact of gender-related abuse across New York transwomen's life course via 2004–2009 longitudinal study. Data gathered from psychometric inventories and interviews using the Life Review of Transgender Experiences (LRTE) to obtain responses for 5 time periods (early/late adolescence, early adult/young adult, early middle age). Figures used here are for the total population's lifetime suicidality
17	Maguen and Shipherd,^29^ Shipherd et al.^30^	153		Ever: 17.65%	Investigated suicidality among participants at a transgender conference in New England known to focus on ‘cross-dressers’ using a questionnaire completed at the conference. Unlike Adams et al.^3^ our analysis includes all respondents, regardless as to whether they answered suicidality questions
18	Effrig et al.,^31^ Hayes et al.^32^	108	Ever: 48.15%	Ever: 25%	Investigated victimization and psychological distress among transgender college students using clinical and nonclinical samples. Clinical sample comprised college counseling center patients where ‘other’ not a gender option. Nonclinical sample consisted of respondents to survey conducted by colleges aligned with counseling centers. Both used the same survey measure, but institutions varied in incentives and completion prompts. Effrig et al.^31^ contains contradictory figures for number and suicidality of nonclinical and clinical participants. We rationalized them to reveal a probable 108 participants in both samples. In a previous article,^3^ a different rate of suicidality was arrived at, because of the use of only participants that answered suicidality questions
19	Meier et al.^33^	431		Ever: 42.69%	Investigated gender confirming hormonal treatment on FTM individuals over 3 months (2008) using primarily US respondents. Suicidality findings were calculated to be accurate to 2 decimal points and therefore differ from Adams et al.^3^
20	House et al.^34^	164		Ever: 34.80%	Explored social and psychological experiences of sexual minorities via internet survey of LGBT respondents conducted over 1 month (2004). 14.6% of respondents were transgender and this group was compared to LGB respondents.
21	Beemyn and Rankin,^35^ Testa et al.^36^	3087	First felt transgender: 16.62%		Explored transgender individuals' life experiences with an online survey conducted over 3 months (2005–2006). Investigated if respondents felt suicidal when they first realized they were transgender and if it was negatively correlated with exposure to positive representations of transgender individuals
22	Heinz and MacFarlane^37^	54	Ever: 35.19%	Ever: 27.78%	Explored health and social service needs of transgender people on Vancouver Island by survey from 2010 to 2011
23	Brown et al.^38,39^	9	Ever: 55.56%	Ever: 11.11%	Investigated life experiences of transfeminine individuals in the Missouri-Kansas City area by semistructured qualitative interviews conducted over an unspecified time period. Suicidality information was spontaneously shared and always in the context of being pretransition
24	Moody and Smith^40^	133^a^	Ever: 65.41%; past year: 74.44%	Ever: 26.32%	Explored suicidality and resilience among transgender Canadians (majority from Quebec and Ontario) through online survey that used verified psychometric inventories
25	Blosnich et al.^41^	1326	2011: 5.13%		Tracked suicidality among transgender veterans with *ICD-9* code indicating transgender-related diagnosis through analysis of US VA EHRs. Records searched from 2000 to 2011, although “suicide related-behaviors” or “events” only available for 2009–2011. Overlapping data were provided for each of these years and 2011 is reported here. a lot of overlap with McDuffie et al.,^27^ Brown et al.,^65^ and Bukowski et al.,^110^ although the way they collected and reported patient data differ
26	Grant et al.,^42,43^ Haas et al.^44^	6456		Ever: 39.92%	Community needs assessment that examined health and social service needs of the transgender population of the US from 2008 to 2009 using a survey conducted primarily online, as well as at survey parties to increase participation of hard to find populations. These figures were calculated using an *n* of 6456, which represents all study participants, in contract with Adams et al.^3^
27	Mereish et al.^45^	16	Ever: 68.75%	Ever: 31.25%	Explored LGBT victimization, substance use, and suicidality among individuals at New England community health center with large LGBT practice. All patients asked to complete a 25-question survey while waiting for physical or mental health appointment over 2 years (2001–2003). Respondents identified gender as male, female, or transgender (1.10% identified as “transgender” and “sexual minority”)
28	Reisner et al. 2014^46^	31	Ever: 58.06%	Ever: 29.03%	Massachusetts community health center research on transgender health disparities using data from patient questionnaires completed before medical appointment during 1 year (2001–2002). Transgender mutually exclusive to male or female and paired with cis gender controls, matched for age (within 3 years), ethnicity, education, and income. Possible crossover with Reisner et al.;^47^ Reisner et al.;^66^ and Beckwith et al.^109^
29	Reisner et al.^47^	23		Ever: 21.74%	Boston community health center study that examined suicidality in a cohort of FTM patients screened for STDs from July to December 2007. Data obtained through a retrospective chart review and past suicide attempts documented in the EHR. Likely some crossover with Reisner et al.^66^
30	Wilson et al.,^48^ Santos et al.,^49^ de Haan et al.^50^	314	Ever: 53.18%		Investigated access to physical, mental health and transition-related health care among transgender women in San Francisco. The data was obtained from a behavioral survey conducted via a handheld computer over 4 months in 2010.
31	Rosser et al.,^51^ Perez-Brumer et al.^52^	1229		Ever: 28.89%; past year: 4.15%	Reports on the social demographics of hidden sexual minorities and transgender-specific individual and structural suicidality risk factors by a sample recruited through an internet-based assessment of US transgender adults. The suicidality figures reported here are slightly different from Adams et al.,^3^ as raw participant figures were previously calculated as fractions
32	Scanlon et al.,^53^ Rotondi et al.,^54,55^ Bauer et al.,^56, 57^ Scheim and Bauer,^58^ Bauer et al.^59^	433	Ever: 77%; past year: 36%	Ever: 43%; past year: 10%	Trans Pulse was a community needs assessment of Ontario transgender health and social service needs conducted from 2009 to 2010 using respondent-driven sampling and data gathered by survey completed online or in person
33	Edelman et al.^60^	521	Ever: 60%	Ever: 34%; past year: 10%	Needs assessment conducted from May 2012 to 2013 that investigated transgender residents of the Washington, DC Metro Area and update of previous study conducted in 2000 (Xavier).^17^ The survey questionnaire was self-administered in most cases
34	Mustanksi et al.,^61^ Liu and Mustanski,^62^ Mustanski and Liu,^63^ Birkett et al.^64^	21		Ever: 52.40%; past year: 19.00%	Explored LGBT youth suicidality (33.3% <18 years), 8.86% of whom identified as MTF or FTM which, because of small size, were collapsed into a single sample. Respondents completed self-report measures and structured and administered psychiatric interviews at initial contact and after 12 months. Data were collected from 2007 to 2011.
35	Brown and Jones^65^	5135	Ever: 19.36%		Reports health disparities in transgender veterans who received VA health care from 1996 to 2013 and whose sex marker changed after enrolment. Suicidality determined through diagnostic code in patient file and transgender and non-transgender veterans compared 1–3. Overlap with McDuffie et al.,^27^ Blosnich et al.,^41^ and Bukowski et al.,^110^ although they differ in data collection and reporting.
36	Reisner et al.^66^	180	Ever: 31.11%	Ever: 17.22%	Assessed mental health among transgender youth (mean age 19.7), seen at Boston-area community health center from 2002 to 2011 using EHRs. Transgender respondents (FTM and MTF) matched to cis gender controls within 3 months of being noted as transgender in file and by gender identity, age, and race/ethnicity. Data used included patient registration, behavioral intake, case notes, and suicidality according to patient self-report. Possible crossover with Reisner et al.;^47^ Reisner et al.;^46^ and Beckwith et al.^109^
37	Olson et al., 2015,^67^ Olson^68^	96	Ever: 51.04%	Ever: 30.21%	Investigated physiological and psychological health among transgender youth (age 12–24, mean 19.2) who presented for gender services at Los Angeles children's hospital (Feb 2011–June 2013). Psychosocial health assessed with computer-assisted self-administered survey and psychometric items and physiologic health from patient files
38	Kuper^69^	1956	Ever: 95.50%; past year: 80.20%	Ever:: 32.30%; Past year: 10.40%	Reported on suicidality and gender development among gender nonconforming youth and young adults from across the United States through an online survey. These findings are slightly different from Adams et al.^3^ because of an error in transcription
39	Weir^70^	5	Ever: 40%	Ever: 20%	Examined impact of violence on trans people and their supports using participants from Alberta and Saskatchewan recruited through online snowball sampling online. Data obtained from administered questionnaires and semistructured interviews. Suicidality data appear to have arisen during interviews
40	Tebbe and Moradi^71^	335^a^	Past year: 71.90%	Ever: 28.10%	Compared US transgender and cis gender college students stress coping strategies using data from national intercollegiate study that distributed surveys to random selection of over 100,000 college students at 73 different institutions in Spring 2011. Transgender cohort consisted of those who identified as neither male nor female. Three participants appeared to be intersex
41	Lehavot et al.^72^	212	Ever: 54.72%; past year: 56.60%	Ever: 32.08%	Examined the life experiences of US transgender veterans recruited online through an anonymous online survey (Feb–May 2014)
42	Grossman et al.^73^	129	Ever: 40.31%	Ever: 22.48%	Explored suicidality among transgender and gender nonconforming youth from three US cities (Nov 2011–Oct 2012) using snowball sampling and an administered survey. Suicidality data obtained through components of Interpersonal Needs Questionnaire
43	Reisner et al.,^74^ Katz-Wise et al.^75^	452		Ever: 32.74%	Investigated change in attraction and social determinants of mental health in Massachusetts transgender adults from August to December 2013 using an online survey
44	Lytle et al.^76^	174	Past year: 31.03%	Past year: 17.82%	Explored transgender college students self-violence and depression with data from US National College Health Assessment survey (Fall 2008–Spring 2009)
45	Dowshen et al.^77^	66	Past year: 15.60%	Past year: 7.80%	Investigated behavioral risk factors and health status of HIV-positive youth using a sample of adolescents and young adults from select US cities. Data were gathered using “computer-assisted self-interviews” from December 2009 to June 2012
46	James et al.^78^	27,715	Ever: 82%; past year: 48%	Ever: 40%; past year: 7%	Compared US transgender and cis gender college students stress coping strategies using data from national intercollegiate study that distributed surveys to random selection of >100,000 college students at 73 different institutions in Spring 2011. Transgender cohort consisted of those who identified as neither male nor female
47	Nemoto et al.,^79^ Operario and Nemoto,^80^ Nemoto et al.,^81^Glynn et al.^82^	573	Ever: 54.97%	Ever: 33.33%	Study undertaken over 8 months (2000–2001) and from 2004 to 2006 that recruited primarily San Franciscan transgender women of color (African American, Latina, Asian/Pacific Islander) with histories of sex work. The research focused on the HIV impact, socioeconomic status, victimization, and physical and mental health
48	Xavier et al.,^83^ Goldblum et al.,^84^ Testa et al.,^85^ Barboza et al.^86^	350	Ever: 63.71%	Ever: 25.43%	Explored the health and service needs of transgender Virginians through online and paper questionnaires administered over 10 months (2005–2006)
49	Trujillo et al.^87^	78		Ever: 38.46%	Reported on beneficial effects of social support on transgender mental health using data obtained from a US national online survey (Feb 2013–April 2014)
50	Brown et al.^88^	11	Ever: 18.18%		An extension of an earlier study completed by the lead author (Brown et al.^38.39^ this publication reports on Mid-Western trans masculine individuals. Suicidality information was garnered from the semistructured interviews
51	Fredriksen-Goldsen et al.,^89^ Fredriksen-Goldsen et al.,^90^ Hoy-Ellis et al.^91^	174	Ever: 71.10%		Explored social and psychological experiences of sexual minorities in an internet survey of LGBT respondents over 1 month (2004). The transgender and lesbian, gay, and bisexual respondents were compared
52	Irwin et al.,^92^ Su et al.,^93^ Brennan et al.^94^	83	Ever: 73.49%	Ever: 44.58%	Community-based participatory research project arising out of a 2010 analysis of LGBT suicidal ideation and health disparities (11.9% transgender, where options were male, female, and/or transgender)
53	Kattari et al.,^95^ Seelman et al.,^96^ Seelman et al.^97^	417	Past year: 34.05%	Past year: 9.35%	Examined health disparities among Colorado transgender adults using a self-completed survey conducted over 7 months in 2014
54	Veale et al.^98,99,100^	600	Ever: 55.67%; past year: 51.00%	Ever: 25.50%; past year: 26.17%	Investigated Canadian transgender youths health using a self-administered online survey conducted from Oct 1, 2013 to May 31, 2014. The older youth (19–25) are reported on here
55	Swanbrow Becker et al.^101^	47	Ever: 55%; past year: 24%	Ever: 34%; past year: 4.30%	This case–control study reported on the stress coping strategies of US transgender college students in comparison with their cis gender peers. Data were collected from a national intercollegiate study that distributed electronic surveys to a random selection of >100,000 college students at 73 different institutions in the Spring of 2011. The transgender cohort consisted of those who identified as neither male nor female. Suicidality was assessed over the life time and “recently” using single questions
56	Testa et al.^102^	816^a^	Ever: 79.20%; past year: 56.10%	Ever: 45.80%	Investigated effects of stress and resilience among transgender and gender nonconforming individuals on mental and physical health using data obtained from Canadian and US adults through online surveys. Seven intersex participants were included in this dataset
57	Bouris and Hill^103^	28	Past year: 17.86%		Chicago study compared gender and sexual minority participants to investigate relationship between youth and their mother figure as means of promoting resilience and exploring minority stress. Participants completed interviewer-administered questionnaire between August 2013 and 2014
58	Lytle et al., 2017^104^	18	Past year: 61.11%		Explored suicidality and help seeking behavior among transgender youth from the Trevor Project's social network using an online questionnaire during August 2012. Individuals who experienced suicidality in past 14 days were excluded
59	Gowin et al.^105^	45	Ever: 55.56%		First attempt to investigate transgender suicidality solely among asylum seekers. Reviewed 2012 US asylum files of Mexican transwomen identified according to stated gender identity or behaviors. Suicidality data garnered from these records
60	Redinger^106^	252		Past year: 11.9%	Recruited transgender Montana High School students to complete a questionnaire on Montana sex education curriculum. Data collected from 2015 to 2016. Respondents were asked to comment on their suicidality in relation to being a high school student.
61	Gaither et al.^107^	330	Ever: 2.12%		Retrospectively reviewed the charts of MTF patients presented for primary GRS to a US high volume surgeon from 2011 to 2015. Patient charts were reviewed pre, intra, and postoperatively for medical morbidities, including a history of suicidality
62	Herman et al.^108^	92,000	Ever: 34%	Ever: 22%	Reports data obtained from telephone interview survey (2015–2016 California Health Interview Survey). Findings extrapolated to statistically valid California-wide estimate of transgender health data
63	Beckwith et al.^109^	145		Ever: 13.79%	Investigated relationship between GRS and suicide using EHRs of binary gendered patients from larger randomly selected sample of transgender patients that presented to at least 1 health care visit to a Boston community health center (July 1, 2010 to June 30, 2015). Possible crossover with Reisner et al.^46,47,66^
64	Bukowski et al.^110^	5072	Ever: 18.36%		Explores health of transgender veterans who received VA treatment from 1997 to 2014 using data from EHRs where records indicated any of 4 *ICD-9* transgender-related diagnostic codes. Suicidality determined from diagnostic code in patient file. Study has quite a bit of overlap with McDuffie et al.,^27^ Blosnich et al.,^41^ and Brown et al.,^65^ although they differ in how they collected and reported on data

In all cases, the y-axis of each figure represents the proportion of the cohort population. The data sets are organized from left to right, by increasing suicidality figure found by each study.

^a^ These studies seem to have included intersex and/or cis gender respondents, without allowing for their suicidality data to be individually assessed.

EHR, electronic health record; FTM, female to male; GRS, gender reassignment surgery, alternatively known as gender affirming surgery; *ICD*, International Classification of Diseases; MTF, male to female; VA, veterans administration.

### Historical trends

As previously noted, the scientific and clinical approach to transgender health has generally evolved from the use of a disease-based model, prevalent from the 1950s to the 1990s,^6,111^ to the one that uses an identity-based lens.^3,112^ Under this lens, transgender SITBs can be understood as significantly being influenced by anti-transgender discrimination and minority stress.^22,28,53^ Within the disease-based lens, however, transgender SITBs are attributed to individualized psychopathology or neurobiology.^8,113^ The literature on transgender health has, therefore, increasingly moved from viewing transgender people as aberrant and pathological, to affirming gender identity as part of a varied spectrum and, where appropriate, offering supportive interventions. In this context, practitioners provide medical and therapeutic interventions that affirm rather than “correct” an individual's gender identity.^114^ Nevertheless, the disease-based lens continues to be employed in some cases.^112,115^

### Meta-analyses and systematic reviews

There are 25 meta-analyses and systematic reviews summarizing the literature on transgender SITBs. However, 20 address the issue primarily among youth or children; as secondary or incidental to another issue (e.g., HIV), a single aspect of a larger topic (e.g., transgender health); or among sexual minorities writ large (e.g., LGBT). The five meta-analyses and systematic reviews that primarily assessed SITBs among transgender adults are Marshall et al.,^116^ Virupaksha et al.,^117^ McNeil et al.,^118^ Wolford-Clevenger et al.,^119^ and Adams et al.^3^ Two^116,118^ account for review parameters (exclusion of gray literature) but fail to identify a number of studies and, in one case, count a single data set multiple times. One^117^ includes gray literature, but only selects articles freely available online and relevant to the review title, without explaining how the latter was systematized. One^119^ identifies factors (attempts, behavior, and deaths) correlated with transgender SITBs in literature published between 1991 and January 2017 but does not analyze data on SITBs in this literature. One^3^ presents an earlier stage of this research, which excludes literature published since February 2016 and does not focus on the impact of ethnicity, income, or education on SITBs. Therefore, this systematic review provides a more robust synthesis and analysis of the available data on transgender SITBs.

## Methodology

### Eligibility criteria

As in the previous publication on this topic,^3^ studies were included in the systematic review if they took place in Canada or the United States; published in English-language journals, theses, or project/institutional reports between 1997 and 2017; reported original participant research on transgender suicide attempts and/or ideation; and included quantitative data on SITBs among ≥5 participants, at least ≥51% of whom were 18 years or older at the time of participation. In addition, this systematic review incorporated studies on both clinical and nonclinical populations (Table 1). The former typically required participants to have a diagnosis of gender dysphoria^120^ and/or be receiving transitional health care services (e.g., hormones and surgery). The latter, by contrast, tended to include participants based on self-identification as any of several transgender identities. Articles were excluded if they reported only on suicide deaths or nonsuicidal self-injury, single case reports, or subsumed transgender data into a larger population (e.g., LGBT). Although research published multiple times was only counted once, all reports were assessed for useful data. Nonwritten reports like poster presentations were excluded. The inclusion of gray literature helped to increase the data pool, reduce the impact of publication bias, and incorporate thesis research that may ultimately result in peer-reviewed publications. Note, however, that only 9%, or 14% of all studies, were non-peer reviewed by the end of the review inclusion period. Given the observed tendency for these publications to achieve peer review over time, it can be assumed that the majority will gain this status, particularly the 6 published since 2015.

### Data collection

Of the 64 distinct research projects (and 108 articles) that make up this systematic review, 7 originated in Canada and 57 in the United States. Where possible we excluded participants <18 years; however, as data on SITBs was usually presented for the totality of participants, this was not always possible. Of these 64 projects, 55 were peer reviewed, 3 were theses, and 6 were project or institutional reports. We then extracted partial or complete data on transgender participants' race/ethnicity (*n*=56), education (*n*=49), and income (*n*=33). In some cases, we supplemented this through interviews with the study authors, which the first author conducted as part of his MSW thesis. Specifically, the first author interviewed 18 authors of publications on transgender SITBs and, in some cases, minor additional statistical information was reported in these interviews. We have not indicated where this occurred to ensure the confidentiality guaranteed in the initial research ethics board approval from Dalhousie University.^121^

### Literature review

This literature review is the culmination of three sequential reviews: the lead author's thesis,^121^ an update^3^ focused on 1997 to February 2016, and the one conducted for this article that focused on 21 years of research on transgender SITBs (1997–2017 inclusive). Literature was identified primarily by keyword searching WorldCat, Google Scholar, and Google Search. WorldCat, as one of the largest catalogs of cross-referenced and multidisciplinary academic literature, was the primary source of material. The following keywords were used: “transgender suicide”; “transsexual suicide”; “FTM suicide”; “MTF suicide”; “transsexual suicidality”; “transgender suicidality”; “transgender suicide attempt”; and “transsexual suicide attempt.” This was supplemented by hand searching the reference lists of identified articles and looking for new publications from previously identified research projects.^3,121^ The latter were identified by searching named projects (e.g., The Trans PULSE Project)^53^ and checking the outputs of identified authors on Google Scholar, Google Search, PubMed, LinkedIn, and ResearchGate. We also reviewed Ramsay's collated list of publications on transgender SITBs.^122^

**FIG. 1.**
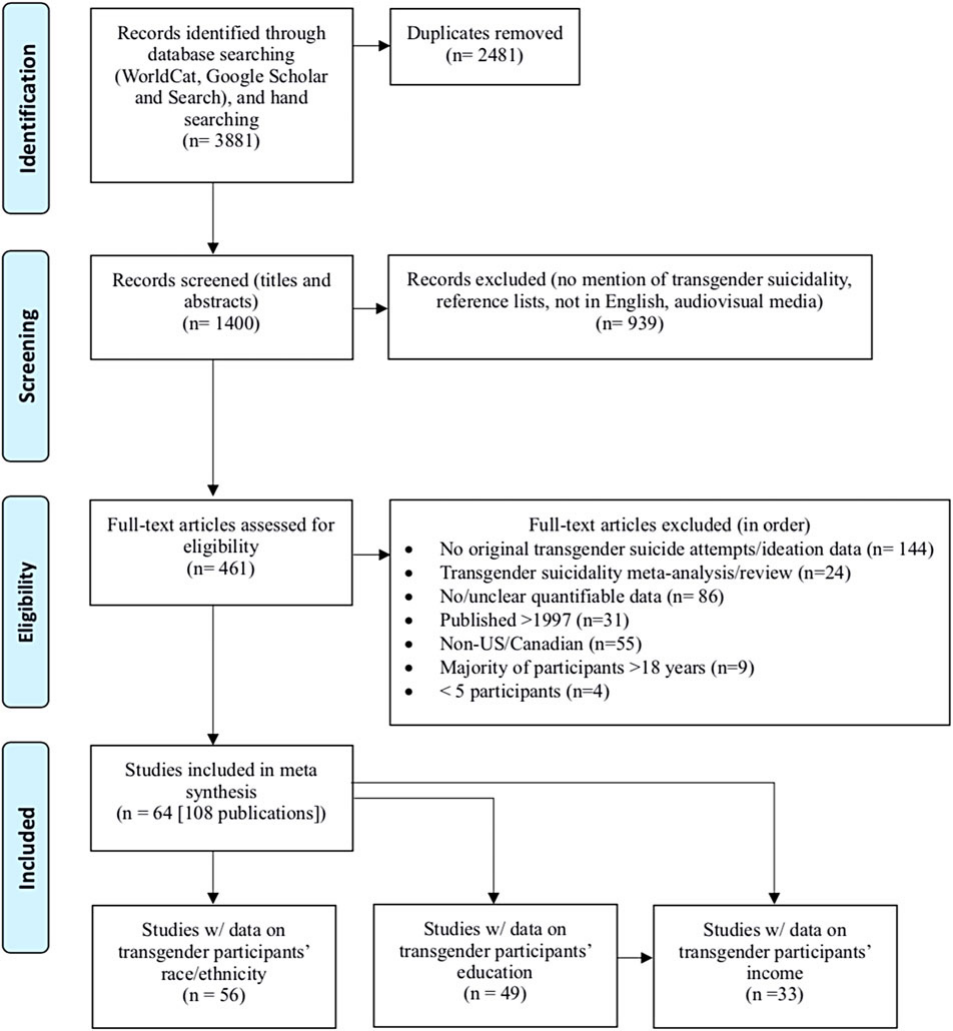
PRISMA Chart.

We initially identified 3,881 articles, of which 2,481 duplicates were eliminated (Fig. 1). Upon reviewing titles and abstracts of the remaining 1,400 articles, we excluded a further 939 that failed to mention transgender SITBs, consisted exclusively of reference lists or audiovisual material, or were not in English. The full texts of the remaining 461 were reviewed and the following eliminations made: reported no original data on transgender suicide attempts and/or ideation (*n*=144), meta-analyses/reviews of transgender SITBs (*n*=24), no/unclear quantitative data (*n*=*n*=86), published before 1997 (31), not from the United States or Canada (*n*=55), the majority of participants were <18 years (*n*=9), and had ≤5 participants (*n*=4). The remaining 108 articles, representing 64 distinct research projects, were included in this systematic review.

### Data analysis

We extracted data on suicide attempts, ideation, and demographic details (race/ethnicity [*n*=56],^ii^ education [*n*=49],^iii^ and income [*n*=33]^iv^) from 64 research projects. Whenever possible we recalculated these data using all study participants as the denominator, rather than only those that answered the given question, positioning nonrespondents as “negative cases' and our findings as conservative. SPSS v. 23 and Microsoft Excel 2016 were used to conduct two analyses. The first examined race/ethnicity, education, and income of transgender participants compared with US Census data. The US Census was used as a baseline because 89% of these projects originated in the US Census and, as a result, most took their demographic categories from it. The second analysis examined lifetime transgender suicide attempt statistics reported according to race/ethnicity and education. Other analyses (income and ideation) of SITBs were excluded for lack of sufficient data.

Of the 56 projects that reported data on race/ethnicity, four (7, 35, 59, 64)^v^ were excluded from further analysis for reporting an amalgamation of ideation and attempts; three (9, 21, 60) for specifying SITBs over a nonstandard time period (e.g., when participants ‘first felt transgender’); six (19, 32, 38, 42, 52, 56) because race/ethnicity data were not collected using mutually exclusive categories, such that the proportions of each racial/ethnic group could not be considered in relation to the study's population size; and five (14, 44–45, 53, 57) for recording SITBs over participant's last year rather than their lifetime. The race/ethnicity data of the remaining 38 projects (*n*=26 for ideation, *n*=35 for attempts) was assessed. Our second race/ethnicity analysis began with the 19 projects that analyzed data on SITBs specific to participants' race/ethnicity. Seven of these (20, 36, 43, 48, 52–53, 56) were excluded because data were reported as a statistical analysis or model, one (42) because ethnic-specific data on SITBs were recorded differently than overall demographic data, and one (44) because SITBs were recorded over a fixed period. The ethnic/race-specific SITBs data of the remaining 10 (6, 11, 19, 26, 31–33, 41, 46–47) projects were compared.

Of the 49 projects that reported on participant educational data, eight (1, 2, 6, 14–16, 28, 52) were excluded for doing so in a manner (e.g., Hollingshead average) inconsistent with the majority, seven (3, 7, 44–45, 53, 57, 60) for recording SITBs during a fixed time, one (59) for conflating suicidal ideation and attempts, and one (18) because being a college student was a requirement. The education data of the remaining 32 projects (*n*=20 ideation, *n*=29 for attempts) were assessed. Our second educational analysis began with the 10 projects that analyzed SITBs with regard to participants' educational level. We eliminated five (20, 41, 43, 48, 52) that reported on this in a statistical model. The educational data of the remaining five (11, 19, 26, 31, 46) were assessed.

Of the 33 projects that reported on participant income, 16 (1, 2, 7–8, 11, 14, 28, 30, 32, 35, 40, 47–48, 53) were eliminated for doing so in a nonstandard format (e.g., relation to poverty line). The income data of the remaining 17 projects (10 for lifetime ideation and 16 for attempts) were assessed. Important to note, however; only five projects provided comparable income data for ideation and seven for attempts using the same five common income ranges from ≤ $10,000 to ≥ $100,000. Our analysis of income was, as a result, limited.

## Results

**FIG. 2.**
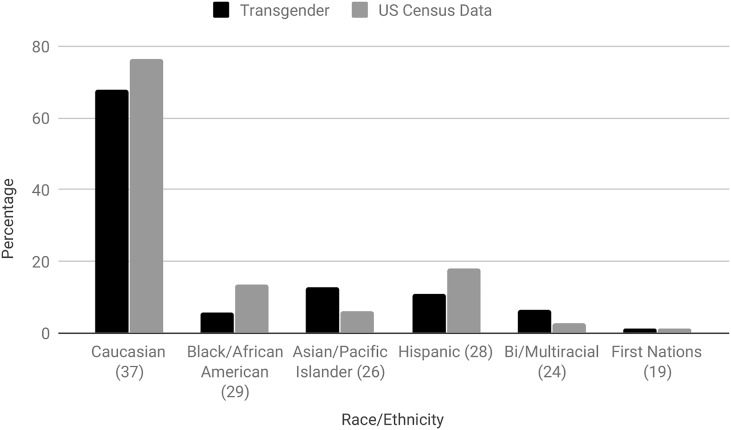
Transgender race/ethnicity versus US Census data. Figures in parenthesis specify the number of individual studies used in the calculation of transgender race/ethnicity.

**FIG. 3.**
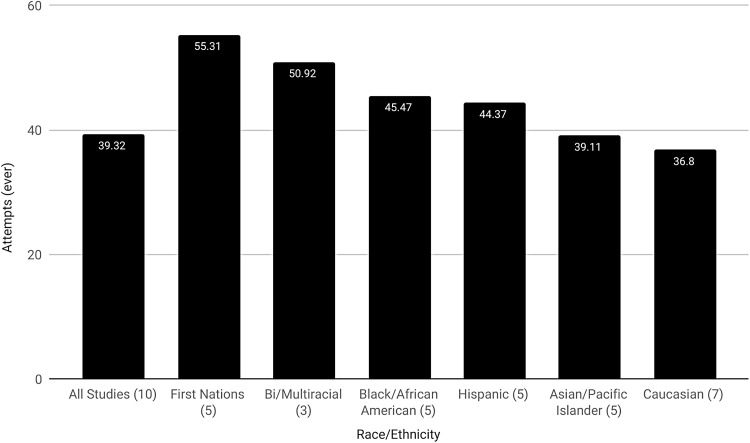
Lifetime transgender suicide attempts according to race/ethnicity. Figures in parenthesis specify the number of individual studies used in the calculation of suicide attempts specific to transgender race/ethnicity.

Lifetime suicidal ideation was reported in 34 studies (average=46.55%; range=18.18–95.50%) and attempts in 46 studies (average=27.19%; range=8.57–52.40%). Average lifetime SITBs in the 38 projects that reported comparable data on both suicide and race/ethnicity was 45.44% for ideation (range=18.18–82%) and 27.88% (0–40.00%) for attempts. An average of 67.75% (3.70–96.73%)^vi^ of these participants were Caucasian and 28.05% (0–95.63%) were nonwhite. Participants in 12.78% (0–21.74%) of the projects were Asian/Pacific Islanders; 11.03% (0–43.90%) Hispanics; 6.57% (0–28%) and 5.58% (0–69.43%) Black/African American and biracial/multiracial, respectively; and 1.46% (0–27.40%) First Nations. A final 0.79% (0.15–18.15%) were classified as “other,” whereas the ethnicity of 4.44% (0–68.52%) was unknown. In comparison with US Census data (Fig. 2),^123^ our findings show fewer Caucasian (67.75% vs. 76.6%), Black/African American (5.58% vs. 13.4%), and Hispanic (11.03% vs. 18.1%), greater Asian/Pacific Islanders (12.78% vs. 6%) and biracial/multiracial (6.57% vs. 2.7%), and roughly equal First Nations participants (1.46% vs. 1.3%). Average lifetime suicide attempts using data on SITBs specific to race/ethnicity (Fig. 3) was 39.32%. First Nations reported the highest rate of lifetime suicide attempts (55.31%), followed by biracial/multiracial individuals (50.92%), Black/African Americans (45.47%), Hispanics (44.37%), Asian/Pacific Islanders (39.11%), and Caucasians (36.80%).

**FIG. 4.**
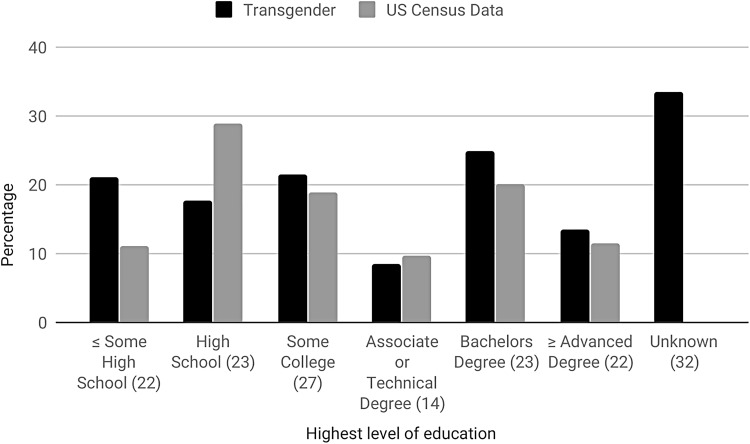
Transgender educational attainment versus US Census data. Figures in parenthesis specify the number of individual studies used in the calculation of transgender educational attainment.

**FIG. 5.**
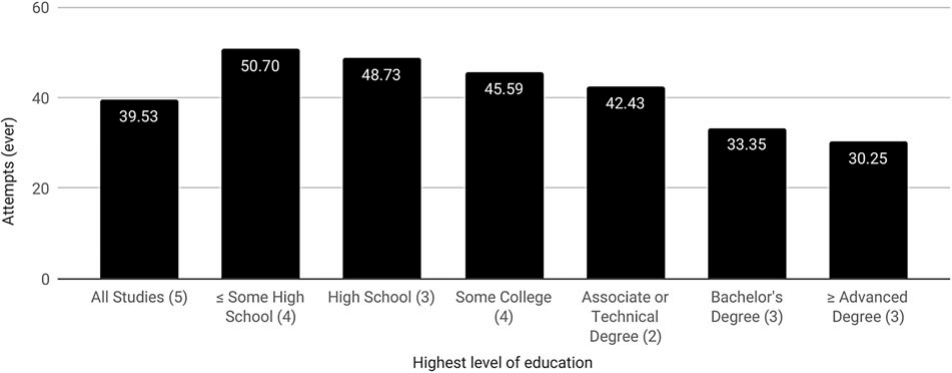
Lifetime transgender suicide attempts according to educational attainment. Figures in parenthesis specify the number of individual studies used in the calculation of suicide attempts specific to transgender educational attainment.

The average rate of lifetime SITBs in the 32 projects that reported comparable data on both suicide and education was 46.26% (18.18–95.50%) for ideation and 27.16% (11.11–43%) for attempts. An average of 24.79% (2.87–36.90%) of participants completed a bachelor's degree, 21.5% (7.32–100%) some college, 21.06% ≤ (0.47–40.08%) some high school, 17.59% (6.40–63.06%) high school, 13.53% (7.32–100%) ≥ an advanced degree,^vii^ and 8.58% (1.15–27.27%) an associate or technical degree. The educational achievement of 33.55% (0–80.80%) of participants was unknown. Compared with the US Census (Fig. 4),^124^ transgender participants had lower rates of high school (17.59% vs. 28.89%) completion and higher attainment of ≤ some high school (21.06% vs. 11.04%), some college (21.5% vs. 18.86%), a bachelor's degree (24.79% vs. 20.04%), and ≥ an advanced degree (13.53% vs. 11.4%). Associate or technical degree attainment was comparable with the general population (8.58% vs. 9.77%). Average lifetime suicide attempts using SITB data on SITBs specific to education (Fig. 5) was 39.53% and appears to drop with the level of education achieved. Specifically, attempts peak among those that had ≤ some high school (50.7%), followed by completed high school (48.73%), some college (45.59%), an associate or technical degree (42.43%), a bachelor's degree (33.35%), and ≥ an advanced degree (30.25%).

**FIG. 6.**
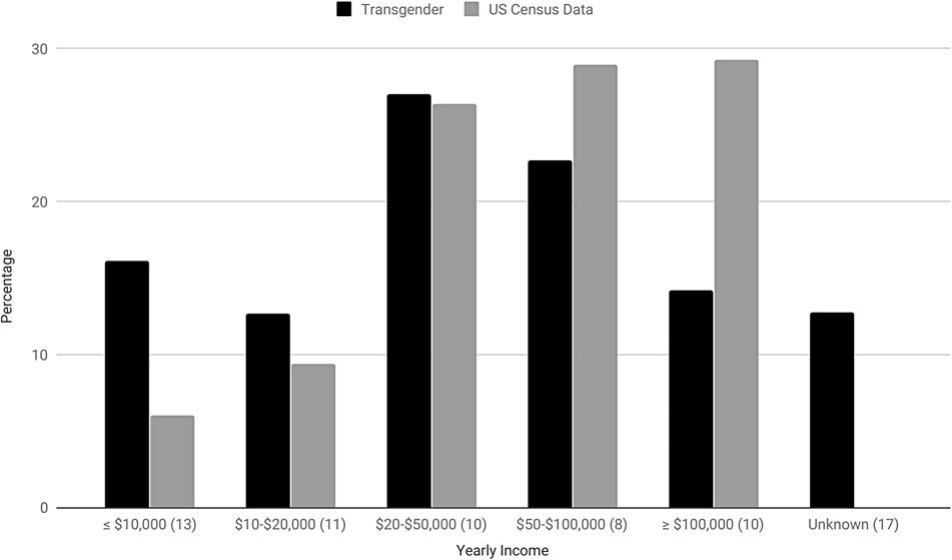
Transgender yearly income versus US Census data. Figures in parenthesis specify the number of individual studies used in the calculation of transgender income.

The average rate of SITBs in the 17 projects that reported comparable data on both suicide and income was 80.76% (34.92–82%) for ideation and 39.25% for attempts (16.27–45.80%). About 26.98% (19.96–40.74%) made $20–50,000, 22.72% (8.45–32.53%) made $50–100,000, 16.14% (8.43–60.32%) made ≤$10,000, 14.17% (2.32–15.21%) made ≥$100,000, and 12.67% (11.68–29.63%) made $10–$20,000. The income of 12.78% (0–89.18%) was unknown. Compared with the US Census (Fig. 6),^125^ transgender participants reported lower overall income. Specifically, more made ≤$10,000 (16.14% vs. 6.05%) and $10–$20,000 (12.67% vs. 9.38%), whereas fewer made $50–$100,000 (22.72% vs. 28.97%) and ≥$100,000 (14.17% vs. 29.25%). The proportion of the transgender and general population making $20–$50,000 was roughly equal (26.98% vs. 26.35%).

## Discussion

### Findings

The methodological rigor of studies on transgender SITBs continues to increase and, although cross-sectional studies are most common, a small but increasing number utilize the more rigorous case–control design. The amount of research on this topic is also increasing, with 14 projects first published from 1997 to 2007 and the remainder since. In fact, following the authors' previous study on this subject, which included studies published to February 2016,^3^ 22 projects, or 34% of the total, have been added. We can expect this trend, which may partially result from the heightened profile of transgender issues and civil rights, to continue. Similarly, although 49 of the studies included fewer than 500 participants, there has been a trend toward exceptionally high cohorts in more recent studies; for instance, *n*=1,250,^51^ *n*=6,456,^43^ *n*=5135,^65^ *n*=27,715,^78^ and *n*=92,000.^108^

Our findings of an average rate of 46.55% lifetime suicidal ideation and 27.19% attempts among transgender participants are similar to those found in the authors' previous study (56% ideation, 29% attempts), although obviously lower with regard to ideation.^3^ The preponderance of evidence suggests that high rates of SITBs, rather than being inherent to transgender individuals, result from barriers to transitional care, stigma, and discrimination.^11,44,59,126^ In addition, by definition, lifetime experience of SITBs is cumulative and while a person may cease to be actively suicidal they will still have had the experience of being suicidal.

It is difficult to make any strong statements on the recruitment and representation of diverse populations in research on transgender SITBs and the relationship between the racial/ethnic diversity of these projects and that of the general population. It is, however, possible to make some basic observations. Online recruitment, although often unable to incorporate the cultural and situational needs of African American participants,^127^ is increasingly seen as an ideal way to access hidden populations like the transgender community.^128^ Employment of key informants and snowball sampling, addressing reluctance to participant in research, and the use of incentives and in person recruitment may help to counter African Americans and other marginalized groups' suspicion of research (p. 3).^129^

Our observation that relatively low rates of suicide attempts occur among Caucasian participants is inconsistent with previous research demonstrating higher rates in this population (Fig. 3).^130^ There is, however, variation in the research literature on this point^131,132^ and evidence exists that capture of Black/African American SITBs, in particular, suffers from data disparities and misclassification.^132,133^ Indeed, we noted relatively high rates of SITBs among Black/African American participants, a group for which SITBs, where police are involved, are sometimes miscategorized as “suicide by cop.”^133,134^ This is one potential explanation for the observation that low rates of SITBs among Black/African American is at odds with other findings of negative comparative race-based health outcomes.^134^ Our findings may suggest that Black/African American transgender individuals are less likely to attempt suicide in a manner that invites misclassification, or possibly that their transgender status itself causes their SITBs to be correctly classified. It should also be noted that we are discussing attempted suicides in which, by definition, the individual has survived and can clarify their intent. However, it should be noted that self-report is not without its limitations, as participants often give different responses to this question depending on the presence of and definition of “attempt” and whether a single question is used.^135^

Our observation of higher rates of SITBs among First Nations individuals^136^ is consistent with other findings that attribute this to colonization-based trauma.^137,138^ Likewise, we observed comparatively low rates among Hispanics and Asian/Pacific-Islanders.^132^ However, there is evidence that SITBs vary greatly within ethnic categories. Puerto Ricans, for instance, have been noted to experience a greater incidence of SITBs than other Hispanics.^132^ Nevertheless, there is comparatively little research on SITBs within racial/ethnic groupings and, depending on the study, conflation of existing categories (e.g., Asian Americans and American Indians). Variable racial/ethnic definitions are also a problem.^132^

Consistent with the rapid increase in biracial/multiracial people in the United States, several studies include information on this population, although few report findings on SITBs specific to them. This is also the case in research on SITBs among nontransgender individuals.^139,140^ Research that does exist tends to focus on youth which, like our own observations, show heightened attempts among biracial/multiracial compared with monoethnic groups.^141^ This may result from experiencing racial discrimination from multiple locations and the stress caused by conflict between one`s internal self and external world.^142^ Nevertheless, the lack of data makes it difficult for us to compare our observations with others and future research into SITBs among biracial/multiracial transgender adults is clearly called for.

Research on transgender individuals' educational attainment is relatively scarce, highly variable, and lacking a clear consensus. As a result, the findings in these projects cannot be presented as typical or generalizable. Comparison of our observations to data from the US Census is made more difficult by variation in the categorization of education within these research projects and with the US Census (Fig. 4). For instance, recent US Census data does not record the category of ‘some high school.’^143^ Nevertheless, the categorization of US Census educational data more closely mirrors the projects in this systematic review than Canadian Census data,^144^ and our own observations grossly compare with the general population. This systematic review also suggests that lower rates of SITBs may be associated with higher levels of educational attainment (Fig. 5), which is largely consistent with other findings of fewer SITBs among individuals with at least a college degree and more among those with less.^145,146^

Many transgender students experience a hostile educational environment that can jeopardize or even disrupt their education. Students so affected may opt out of or be refused access to education because of, for instance, their gender presentation.^147,148^ Although a number of transgender individuals return to school and achieve advanced education later in life, it can be difficult after early and often traumatic disruption,^43^ which may explain some findings of low educational achievement.^147,149^ Given the high number of convenience samples, this may particularly be the case in research that is overrepresented by especially vulnerable participants.^148^ Addressing gender diversity in education and increasing scholarship on this subject will help to challenge the disparities that prevent transgender people from full educational participation and, given the protective effect of educational achievement, may lead to a reduction in their experience of SITBs.^150^

The lack of individual project data on transgender SITBs according to income, and a complete absence of research into lifetime income among transgender individuals, limits our ability to comment on the relationship between these factors. It should also be noted that, although this systematic review found a lifetime suicide ideation rate of 80.76%, it also included a single study that had 27,715 participants and high suicidal ideation. We did, however, observe that several studies noted a relationship between transgender SITBs and high rates of poverty or un/underemployment and,^43,151^ within the literature, transgender individuals were observed to be four times more likely than the general population to make <$10,000/year.^44,151^ This is consistent with the observations of our systematic review that transgender participants are almost three times more likely to make ≤$10,000 (Fig. 6). Similarly, transgender unemployment, discrimination, and poverty are also strongly associated with workplace discrimination and, in turn, lack of education and self-harm.^114^ Another key consideration is that poverty curtails a transgender individual's access to medical transition, which is a known protective factor.^59^ This is especially true in environments, like the United States and some parts of Canada, without transgender-inclusive universal health care.

### Implications for future research

The myth that transition leads to SITBs continues to be used to deny transgender health care access and legislative rights, despite a lack of empirical support for this position and repeated research debunking it.^3,152–159^ A more productive avenue for addressing the phenomenon of transgender SITBs might be to investigate the protective factors inherent in transgender health care,^33,160–163^ and the negative impacts of denied access.^40,57,126^ In particular, research is needed on the impact of resiliency, minority stress,^164^ and culturally protective factors on transgender SITBs. An exploration of syndemic factors, particularly with regard to interrelated issues like racism, poverty, and education, would also be a fruitful avenue for research. For instance, only one study has reported on SITBs among transgender refugees.^105^ We recommend that researchers explore transgender SITBs among various ethnicities, rather than flattening it into a comparison between whites/Caucasians and people of color, ethnic minorities, or racialized individuals. Methods of gathering these data include asking participants to write in their race/ethnicity, reporting on ethnicity and race separately,^124^ using census categories, and/or allowing for the selection of multiple ethnicities.^165,166^ Regardless, the choice should be clearly explained and rationalized in the resulting publications.

We further recommend that, where practical, participants be allowed to write in their actual or estimated income, which will allow comparison across a variety of monetary brackets. Where participants are asked to self-report SITBs, we suggest that a definition be provided of the item being measured and, where possible, multiple questions be asked.^135^ Finally, the creation of project websites will allow findings to be collected and disseminated from a central location. In revising the literature review from the previous publication,^3^ these websites were invaluable in helping us identify project updates and data published across multiple studies. Giving the project a unique and distinct name, ideally with an available website URL, and using it in promotion and branding will assist in making them easily locatable (e.g., The Trans PULSE Project^53^).

### Limitations

These findings are preliminary and lack generalizability for several reasons. The included studies rely heavily on self-reported SITBs and individual definition of “attempt” and “ideation” can vary a great deal, particularly when a definition is not provided and only a single question is used.^135^ Second, because the majority of the 64 projects that make up this systematic review use convenience samples, which do not tend to result in representative or easily replicable data.^147^ There is also a high degree of heterogeneity between these projects; for instance, the categorization of race/ethnicity, education, and income and the size of cohorts (*n*=5–92,000 participants), the latter of which obscures the impact of small samples and overemphasizes the representativeness of large ones. Studies 46 (*n*=27,715) and 62 (*n*=92,000) have a particularly disproportionate impact on the results presented here.^167^ Another concern is the small-study effect, whereby small cohort studies of lower methodological rigor report different effects than larger ones.^167,168^ To some degree, concerns of heterogeneity are mitigated by aggregating these projects into a systematic review, although caution should still be taken in interpreting and applying findings. Indeed, the fact that this is a systematic review is itself limiting, as it is bound to exclude unpublished results because of publication bias,^167^ although this is also mitigated by our inclusion of gray literature.

In addition, income, educational attainment, and race/ethnicity are also often correlated. For instance, one's race/ethnicity directly impacts their income and access to education.^169^ It is, therefore, difficult to observe or discuss these factors in isolation of one another and our findings should be interpreted in light of this limitation. We suggest that future research on the subject of transgender SITBs, in relation to race/ethnicity, education, and income, collect discrete data on each of these factors and subject them to statistical tests, like stepwise regression, to determine the impact of each factor alone and together on SITBs.

Participation bias, particularly in mental health research, is also an issue in recruitment and reporting of participants.^170^ Other research has demonstrated that men and individuals with a substance abuse or dependence history are more likely to participate in research on suicide attempts.^171^ More broadly, people who have experienced SITBs, or are in greater distress generally, may be more likely to participate in studies on this subject. Likewise, research undertaken on clinical populations, or only those with specific presenting issues, may find higher rates of SITBs than population or community-based samples. This is mitigated somewhat by our inclusion of both clinical and nonclinical samples, the latter of which often used population and community-based recruitment. On the contrary, stigma related to SITBs and/or gender identity may prevent individuals from participating in or accurately reporting on their experiences in research on this subject.

As noted in the lead author's previous study,^3^ gender identity definitions and terms are in flux,^172^ differ among individual projects, and documentation of nonbinary genders continues to be limited.^173^ The way individual projects inquire into SITBs is also a concern. It has, for instance, been noted that yes/no questions like “have you ever attempted suicide” tend to overestimate SITBs by combining self-harming behavior with and without suicidal intent (p. 3).^44^ There is some evidence that in-person interviewing reduces reports of attempts among adults, although this is an uncommon methodology within research on transgender SITBs, possibly because of cost and privacy concerns.^3,174,175^

## Conclusion

This systematic review supports findings of high, if heterogeneous and widely variant, rates of SITBs among transgender adults in the past 21 years. With regard to transgender individuals, it observes that Caucasians experience lower rates of SITBs than other groups; that high levels of educational achievement seem to protect against SITBs; and that they may be at risk of higher SITBs because of relatively low income. Research on the topic of transgender SITBs would be improved by greater consistency in data collection and improved research methodology.

## Abbreviations Used

FTM: female to male
MTF: male to female
SITB: suicidal thoughts and behaviors

## Notes

^i^ Projects were included dependent on their last publication on the data set. Devor^5,6^ was the only project to occur partially outside of the 20-year window.

^ii^ The projects not reporting any race/ethnicity data were 1, 3–4, 25, 27, 34, 58, 61.

^iii^ The projects not reporting any educational attainment data were 4, 21, 25, 27, 34–37, 42, 54, 56, 58, 61, 63–64.

^iv^ The projects not reporting any income data were: 3–4, 9, 15–18, 21, 23, 25, 27, 29, 34, 36–39, 41–42, 44–45, 49–50, 54–55, 57–61, 64.

^v^ All bracketed numbers are taken from Table 1.

^vi^ Figures for ethnicity/race, education, and income do not add to 100%, because of differences in the manner that projects classified racial/ethnic categories and because they are not always mutually exclusive.

^vii^ The category of advanced degree consists of at least graduate school or a professional degree.
